# Silent Metastasis of Myxoid Liposarcoma to the Heart Unveiled by Multimodal Imaging

**DOI:** 10.7759/cureus.81727

**Published:** 2025-04-04

**Authors:** Hamza Retal, Imad Bougrine, Soumya El Graini, Tarik Salaheddine, Jamal El Fenni

**Affiliations:** 1 Radiology Department, Ibn Sina University Hospital, Rabat, MAR; 2 Radiology Department, Mohammed V Military Hospital, Rabat, MAR

**Keywords:** cardiac metastasis, cardiac mri, echocardiography, multimodal imaging, myxoid liposarcoma

## Abstract

We present the case of a 58-year-old male with a history of smoking and hypertension, who was diagnosed with and treated for a myxoid liposarcoma of the right thigh. After undergoing surgical resection with negative margins and adjuvant radiotherapy, the patient remained asymptomatic. During a scheduled two-year routine imaging follow-up, a suspicious mass was identified in the right ventricle, incidentally detected on a surveillance scan without preceding clinical symptoms, leading to the diagnosis of metastatic cardiac myxoid liposarcoma. Multimodal imaging, including echocardiography, CT scan, and cardiac MRI, provided crucial insights into the tumor’s characteristics. CT detected the lesion and its anatomical localization. MRI confirmed its lipomatous composition and helped exclude differentials. Echocardiography assessed intracardiac involvement and hemodynamic impact. Surgical resection of the cardiac mass confirmed the diagnosis after histopathologic examination. Despite successful treatment, the patient later died from a myocardial infarction secondary to coronary artery disease, which was not linked to the tumor or surgical events. This case highlights the rarity and diagnostic challenges of metastatic cardiac liposarcoma and emphasizes the role of advanced imaging and surgical intervention in its management.

## Introduction

Liposarcomas are malignant soft tissue tumors originating from adipocytic differentiation, commonly affecting the extremities and retroperitoneum. Myxoid liposarcoma predominantly metastasizes to the lungs and other extrapulmonary sites, such as the bone, serosal surfaces, and soft tissues. Its metastases are primarily hematogenous, likely due to the rich vascular network within the myxoid stroma, which facilitates tumor cell dissemination. Cardiac metastases from liposarcoma are exceedingly rare, with only a few cases reported in the literature. When present, clinical manifestations can vary widely, including arrhythmias, heart failure, or embolic events, depending on the location and extent of myocardial involvement. In some cases, the patient may remain asymptomatic. For instance, prior reports have documented cases where patients presented with ventricular tachycardia as the initial manifestation of metastatic cardiac involvement. Given its rarity and nonspecific presentation, cardiac metastatic liposarcoma poses a diagnostic challenge, necessitating advanced imaging modalities such as echocardiography and cardiac MRI. In particular, fat-suppressed sequences help differentiate lipomatous components, suggesting a lipoma or liposarcoma rather than a myxoma or other non-lipomatous metastatic lesions. Late gadolinium enhancement (LGE) aids in assessing tumor viability and fibrosis and helps distinguish it from differentials such as intracardiac thrombosis [1].

## Case presentation

A 58-year-old male patient with a history of smoking (20 pack-years over 30 years) and essential hypertension managed with monotherapy was initially diagnosed with a superficial myxoid liposarcoma in the right thigh, which was successfully resected. Given the known risk of recurrence and metastasis associated with myxoid liposarcomas, a meticulous surgical approach was employed to ensure complete resection with clear margins. The surgery aimed to remove all visible tumor tissue. Postoperatively, the patient received radiotherapy to minimize the risk of local recurrence. A total dose of 50 Gy was delivered to the surgical site in 25 fractions, targeting the resection margins and surrounding tissues at risk of microscopic disease. The rationale for radiotherapy in this case was based on the high recurrence risk associated with the myxoid liposarcoma subtype, which tends to involve more extensive soft tissue and can spread to adjacent structures. Chemotherapy was not indicated, as the liposarcoma was resected with negative margins and there were no signs of systemic involvement.

**Figure 1:**
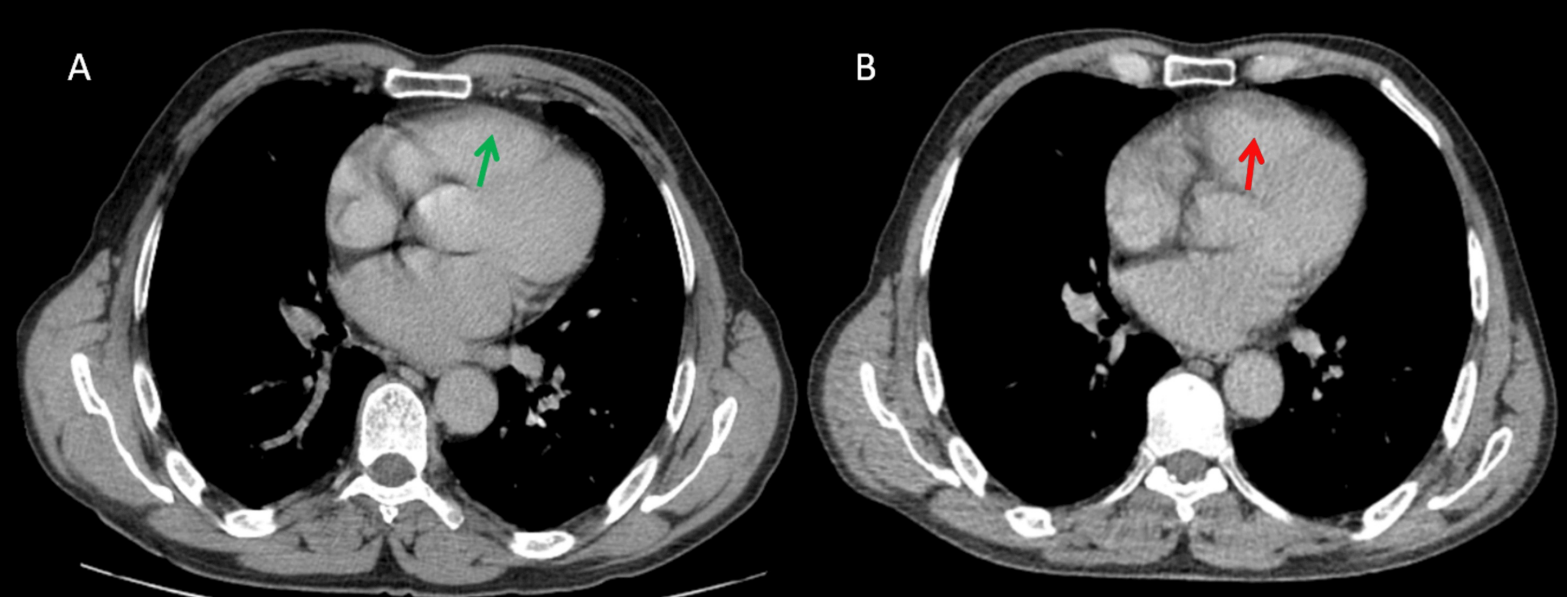
Axial CT scan slices at the level of the cardiac chambers. (A) One-year post-surgical follow-up CT scan demonstrating no cardiac abnormalities, with a preserved right ventricular wall (green arrow). (B) Two-year follow-up CT scan showing an ill-defined hypodense tissue thickening of the right ventricular wall, raising suspicion of a pathological process but remaining non-specific (red arrow).

Two years after the resection, the patient, who remained asymptomatic, underwent a routine follow-up CT scan as part of the standard surveillance protocol. The scan revealed a suspicious isolated 10 mm thickening of the anterior wall of the right ventricle, an abnormality not seen in the previous year's CT scan study (Figure 1).

**Figure 2:**
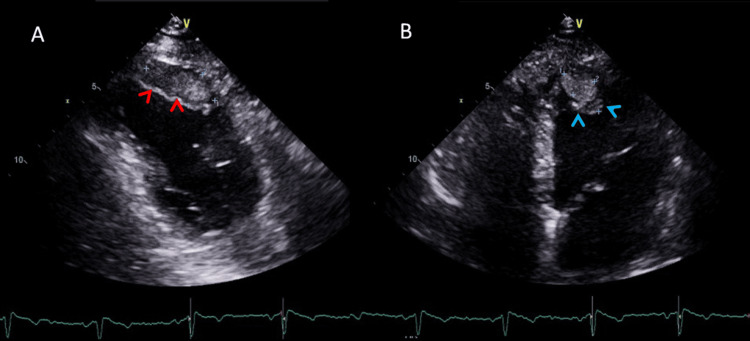
Ultrasound images of the cardiac chambers. (A) Parasternal view of the right ventricle showing an echogenic, slightly heterogeneous intramyocardial mass measuring approximately 17 × 36 mm (red arrowheads). (B) Four-chamber view revealing a second myocardial mass with similar radiologic characteristics, appearing as a slightly heterogeneous echogenic lesion within the apical aspect of the left ventricle, measuring approximately 15 × 23 mm (blue arrowheads).

The mass was incidentally detected during this imaging, with no clinical symptoms leading to its discovery. This raised concerns about potential metastatic involvement. A subsequent cardiac ultrasound (US) was performed, revealing a mass within the myocardium, which further suggested a neoplastic origin. The ultrasound showed two heterogeneous, echogenic masses within the anterior aspect of the right ventricle and the apical aspect of the left ventricle, with no hemodynamic consequence, no impact on ventricular ejection volume, and no effect on myocardial contraction, prompting further evaluation (Figure 2).

**Figure 3:**
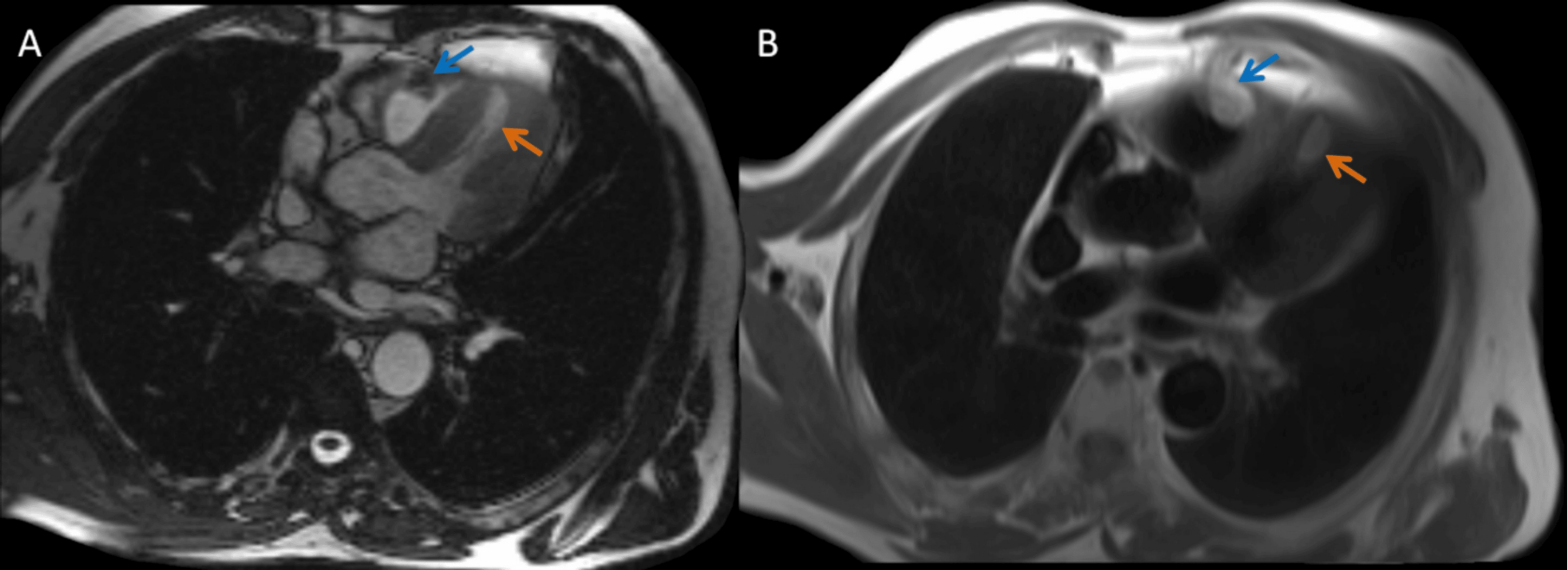
Cardiac MRI sequences: steady-state free precession (SSFP) cine MRI sequence (A) and T1-weighted black blood MRI sequence (B). (A) A bright blood signal. A well-defined, heterogeneous mass is observed in the anterior aspect of the right ventricle (blue arrow). A second lesion is noted within the cardiac apex of the left ventricle presenting with a bright hyperintense signal similar to the surrounding fat (orange arrow). (B) The right (blue arrow) and left (orange arrow) ventricular masses remain visible and exhibit a bright hyperintense signal similar to the surrounding fat. The black blood technique suppresses blood flow, improves visualization of the myocardium and mass effect, and well-defined lesion borders.

To better characterize the mass and assess its precise location, a cardiac MRI was conducted, which revealed a mass showing a high signal intensity on T2-weighted images, a typical feature of myxoid liposarcomas due to their myxoid stroma (Figure 3).

**Figure 4:**
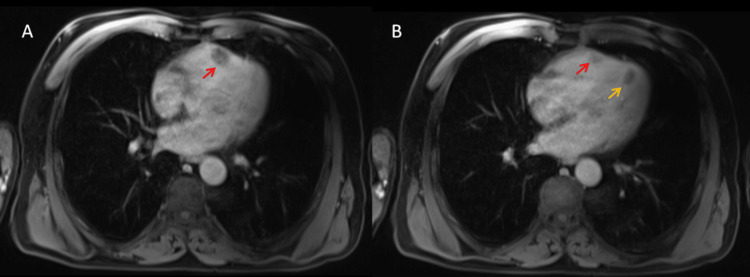
Axial T1-weighted fat-saturated sequence with gadolinium contrast from a cardiac MRI. (A) The right ventricular mass, situated within the anterior myocardial wall, exhibits signal suppression, confirming its fatty composition (red arrow). (B) The left ventricular apical mass shows a similar suppression pattern, further supporting its adipose nature (orange arrow).

On post-contrast imaging, the mass showed heterogeneous enhancement, a characteristic finding in liposarcomas due to their variable vascularity and necrotic components. Fat-suppression sequences confirmed the presence of fatty tissue within the mass, strongly suggesting a lipomatous tumor (Figure 4).

These imaging findings raised the suspicion of metastatic disease originating from the previously resected myxoid liposarcoma. An electrocardiogram (ECG) was conducted to evaluate potential cardiac involvement or effects. The examination revealed normal sinus rhythm, with no signs of arrhythmia, conduction disturbances, or other electrical abnormalities. This suggests that there was no apparent direct impact on the heart's electrical activity related to the mass effect.

Given the high risk of metastasis and cardiac involvement, a multidisciplinary team decided to proceed with surgery. Due to logistical reasons, the operation took place six months after the mass was first detected. The mass was carefully removed from the myocardium, and the tissue analysis confirmed a metastatic myxoid liposarcoma. The pathology report showed the characteristic features of liposarcoma, including mature adipose tissue intermixed with myxoid stroma, slightly increased cellularity, and occasional mitotic figures. Unfortunately, pathology images were not available, as the patient chose to have the pathology examination performed in a different city from his place of origin.

Following surgical resection, the patient was closely monitored during recovery, with no immediate complications. Since there was no evidence of widespread metastatic disease at the time of surgery, the management strategy focused on regular imaging to detect any potential recurrence or metastasis. After a year and a half without recurrence or symptomatic disease, the patient passed away due to a myocardial infarction caused by a coronary event.

## Discussion

Liposarcoma is one of the most prevalent soft tissue sarcomas, accounting for approximately 8-17.8% of cases. It is classified into well-differentiated, dedifferentiated, myxoid, pleomorphic, and mixed subtypes. Among these, myxoid and dedifferentiated variants exhibit a greater propensity for metastasis, frequently targeting the lungs and, less commonly, extrapulmonary sites such as the heart [1]. However, cardiac metastases from liposarcoma are exceedingly rare; only a few cases have been reported in the literature. A comprehensive review identified 47 reported cases of liposarcoma metastasizing to the heart [2,3].

The diagnosis of metastatic cardiac liposarcoma remains challenging due to its often nonspecific or asymptomatic presentation, as observed in our patient, which can lead to delayed detection. When symptomatic, clinical manifestations depend on tumor location and myocardial involvement. Dyspnea and exertional intolerance commonly result from mass effect on the cardiac chambers, while chest pain resembling angina may occur due to coronary artery compression. Arrhythmias, such as atrial fibrillation or ventricular tachycardia, can arise from direct myocardial invasion. Heart failure signs such as lower extremity edema, jugular venous distension, and pulmonary congestion are frequently observed. Additionally, pericardial effusion, with or without tamponade, may develop, particularly in cases with pericardial infiltration [4].

The accurate diagnosis of metastatic cardiac liposarcoma requires a multimodal imaging approach. Echocardiography, often the first-line imaging modality, may reveal a hyperechoic, heterogeneous mass, with or without associated pericardial effusion. It plays a crucial role in distinguishing a mass from thrombi with contrast injection. Computed tomography (CT) typically demonstrates a heterogeneous soft tissue mass, with areas of fat attenuation in well-differentiated liposarcomas, while dedifferentiated and myxoid subtypes appear as non-adipose soft tissue lesions [5]. Magnetic resonance imaging (MRI), offering superior tissue characterization, plays a key role in differentiating liposarcoma metastases from other cardiac neoplasms. Myxoid liposarcomas commonly exhibit high signal intensity on T2-weighted sequences and intermediate signal on T1-weighted images. Fat suppression techniques help confirm adipose tissue components, reinforcing the diagnosis [6,7]. Positron emission tomography-computed tomography (PET-CT) provides additional insight into metabolic activity, as cardiac metastases typically show fluorodeoxyglucose (FDG) avidity, and helps identify other metastatic sites. It is mostly useful at the end of treatment to detect a high metabolic lesion suggestive of residual or recurrent disease, or in the case of an inconclusive MRI. However, cardiac PET has limitations, including radiation exposure, preparation requirements, variability in physiological myocardial uptake, limited availability, and challenges in differentiating primary from metastatic cardiac masses. In addition, inflammatory or infectious conditions, as well as factors such as glucose levels or insulin therapy, can impact PET diagnostic accuracy [8]. The diagnosis of cardiac liposarcoma relies on a combination of histopathological and immunohistochemical (IHC) findings. IHC markers, particularly MDM2 and CDK4, are positive in well-differentiated and dedifferentiated liposarcomas. While these markers are specific for liposarcoma, additional markers such as DDIT3 (specific for myxoid liposarcoma) or p16 (seen in dedifferentiated sarcomas) can help distinguish it from histological mimics, including other myxoid tumors and poorly differentiated sarcomas [9].

Previous reports have emphasized the critical role of imaging in identifying and characterizing cardiac metastases. However, differentiating liposarcomas from other cardiac tumors can be challenging, as these variants may present as soft tissue density lesions. MRI provides superior tissue characterization, which is essential in distinguishing liposarcoma metastases from other cardiac tumors. Post-contrast imaging frequently reveals heterogeneous enhancement, reflecting the tumor’s variable vascularity and necrotic components [4].

The differential diagnosis for a cardiac mass is broad, encompassing both benign and malignant conditions. Among benign cardiac tumors, myxomas are the most common, typically originating from the interatrial septum, most frequently in the left atrium. On echocardiography, myxomas often appear mobile and may demonstrate a "ball-valve" effect, obstructing blood flow through the atrial or ventricular chambers. They are generally homogeneous and echogenic with an irregular surface and are frequently attached by a stalk, a feature not seen in liposarcomas. In contrast, liposarcomas tend to be larger, more infiltrative, and less mobile, with a more heterogeneous texture on imaging. On MRI, myxomas appear as well-defined, non-fatty masses with intermediate signal intensity on T1-weighted images and high signal intensity on T2-weighted images, reflecting their mucinous content. Fat suppression techniques can confirm the absence of fat in myxomas, distinguishing them from liposarcomas, which contain fat components visible on imaging [8,10].

Among malignant tumors, angiosarcomas are another important differential diagnosis, typically involving the right atrium or pericardium. On echocardiography, these tumors appear as large, infiltrative masses with irregular borders. They tend to invade blood vessels, leading to obstruction or thrombosis. MRI typically shows heterogeneous enhancement, but unlike liposarcomas, they lack distinct fat attenuation. The vascular pattern often exhibits irregular, heterogeneous enhancement, distinguishing it from the more fatty, well-defined regions seen in liposarcomas [8,11].

Metastatic cardiac involvement from primary malignancies such as lung cancer, breast cancer, melanoma, and renal cell carcinoma is more common than primary cardiac malignancies. These secondary tumors usually invade the myocardium or pericardium and lack the fatty components characteristic of liposarcomas. On echocardiography, metastatic tumors often present as ill-defined masses, whereas liposarcomas tend to be more localized and well-demarcated. MRI findings of metastatic tumors typically reveal heterogeneous enhancement with areas of necrosis or hemorrhage but without the characteristic fatty density of liposarcomas. Moreover, metastatic tumors often exhibit a more aggressive growth pattern, involving multiple cardiac chambers or extending into the pericardium, whereas liposarcomas are generally more localized and confined to the myocardium or epicardium [8].

A thrombus in the cardiac chambers may also mimic a neoplastic mass, particularly when located in regions of abnormal blood flow, such as the left atrium or right atrial appendage. Unlike tumors, thrombi are typically non-enhancing on contrast imaging. On echocardiography, thrombi appear as homogeneously hypoechoic or anechoic masses attached to the endocardial surface and typically lack contrast enhancement in ultrasound. Delayed contrast-enhanced MRI is crucial, as thrombi typically lack enhancement, whereas liposarcomas show heterogeneous enhancement reflecting viable tumor components. This distinction is particularly important given that thrombi are much more common than primary cardiac sarcomas. Additionally, thrombi do not demonstrate the high signal intensity on fat-saturated MRI sequences that would suggest liposarcoma. The absence of fatty tissue is a key distinguishing feature, as thrombi generally have uniform signal intensity without the complex heterogeneity observed in liposarcomas [8].

Management of metastatic cardiac liposarcoma depends on several factors, including tumor burden, the presence of systemic disease, and the patient’s overall fitness. Surgical resection is the preferred treatment when feasible. Feasibility is primarily determined by tumor location, extent of myocardial infiltration, and proximity to critical cardiac structures such as coronary arteries and valves. Complete resection may be challenging due to the risk of compromising cardiac function. Challenges include recurrence rates, the risk of incomplete resection, and the need for advanced surgical techniques such as autotransplantation in extensive cases. Reconstructive techniques, such as bovine pericardial patch repair, may be required, particularly when significant myocardial tissue must be excised. Intraoperative strategies may include performing a ventriculotomy to remove pathological tissue fused to the myocardium, followed by myocardial reconstruction. Additionally, cryoablation can be utilized to treat tumor-infiltrated margins after resection. Despite the complexity of these interventions, successful surgical management can lead to favorable outcomes, with patients demonstrating good ventricular function postoperatively [12]. Chemotherapy remains the cornerstone treatment for inoperable or metastatic liposarcomas. Anthracycline-based regimens are the first-line option, while trabectedin has shown particular efficacy in myxoid liposarcoma by targeting the FUS-CHOP fusion protein. Eribulin, on the other hand, has demonstrated greater effectiveness in dedifferentiated subtypes. Meanwhile, ongoing clinical trials are investigating novel therapeutic strategies, including tyrosine kinase inhibitors and immunotherapies designed to modulate the tumor microenvironment [13]. Radiotherapy can be utilized in cases with unresectable disease to provide local tumor control or as an adjuvant therapy post-resection if margins are inadequate. Supportive care, including management of heart failure symptoms with diuretics, beta-blockers, and ACE inhibitors, is important. Pericardiocentesis may be required in cases with significant pericardial effusion and tamponade risk [4,14].

Prognostic indicators for metastatic cardiac liposarcoma include tumor size, presence of extracardiac metastases, and response to systemic therapy. Median survival for patients with cardiac primitive sarcomas varies based on factors such as resectability and metastatic spread. Studies have reported median survival times ranging from 6 to 12 months without treatment, extending to 17 to 24 months with complete surgical resection. The prognosis for metastatic liposarcoma involving the heart remains poor, with a significant proportion of patients succumbing within the first year of diagnosis, based on some case reports. However, survival varies widely depending on factors such as the presence of extracardiac metastases and the feasibility of surgical intervention [3,15].

In conclusion, metastatic cardiac liposarcoma is a rare but clinically significant condition that requires a multimodal imaging approach for accurate diagnosis. Imaging plays a critical role in differentiating liposarcoma metastases from other cardiac tumors, and histopathology remains the gold standard for confirmation. Given the rarity of this condition, further studies and case compilations are needed to establish clearer imaging criteria and optimal management strategies. Moreover, as the prognosis is often poor due to the advanced stage at diagnosis, early identification and appropriate treatment remain essential in improving patient outcomes.

## Conclusions

Metastatic cardiac liposarcoma is rare and often difficult to diagnose, primarily due to its nonspecific imaging features and frequently silent clinical course. This case highlights the importance of multimodal imaging, particularly cardiac MRI, in detecting and characterizing cardiac metastases. Key imaging findings, such as fatty tissue composition and enhancement patterns, play a crucial role in distinguishing these tumors from other cardiac masses. While surgical resection remains an option for isolated metastases, the overall prognosis is poor due to late-stage presentation and the aggressive nature of the disease. Further research is needed to refine imaging techniques for earlier detection by implementing a structured follow-up protocol and exploring advanced modalities such as perfusion imaging and parametric mapping, as well as investigating new therapeutic strategies to improve patient outcomes.
